# T‐type calcium channel blockade induces apoptosis in C2C12 myotubes and skeletal muscle via endoplasmic reticulum stress activation

**DOI:** 10.1002/2211-5463.12965

**Published:** 2020-09-15

**Authors:** Mao Chen, Suting Li, Menglei Hao, Jue Chen, Zhihan Zhao, Shasha Hong, Jie Min, Jianming Tang, Ming Hu, Li Hong

**Affiliations:** ^1^ Department of Gynecology and Obstetrics Renmin Hospital of Wuhan University China

**Keywords:** apoptosis, endoplasmic reticulum stress, skeletal muscle wasting, T‐type calcium channel

## Abstract

Loss of T‐type calcium channel (TCC) function has been reported to result in decreased cell viability and impaired muscle regeneration, but the underlying mechanisms remain largely unknown. We previously found that expression of TCC is reduced in aged pelvic floor muscle of multiple vaginal delivery mice, and this is related to endoplasmic reticulum stress (ERS) activation and autophagy flux blockade. In the present work, we further investigated the effects of TCC function loss on C2C12 myotubes and skeletal muscle, which is mediated by promotion of ERS and ultimately contributes to mitochondrial‐related apoptotic cell death. We found that application of a TCC inhibitor induced mitochondria‐related apoptosis in a dose‐dependent manner and also reduced mitochondrial transmembrane potential (MMP), induced mito‐ROS generation, and enhanced expression of mitochondrial apoptosis proteins. Functional inhibition of TCC induced ERS, resulting in disorder of Ca^2+^ homeostasis in endoplasmic reticulum, and ultimately leading to cell apoptosis in C2C12 myotubes. Tibialis anterior muscles of T‐type α1H channel knockout mice displayed a smaller skeletal muscle fiber size and elevated ERS‐mediated apoptosis signaling. Our data point to a novel mechanism whereby TCC blockade leads to ERS activation and terminal mitochondrial‐related apoptotic events in C2C12 myotubes and skeletal muscles.

## Graphical abstract

Loss of T‐type calcium channel (TCC) function has been reported to result in decreased cell viability and impaired muscle regeneration. In this study, we report a novel mechanism whereby TCC blockade promotes ERS and contributes to Ca^2+^ homeostasis disorder of endoplasmic reticulum, thereby resulting in mitochondrial‐related apoptotic cell death in C2C12 myotubes and skeletal muscle.

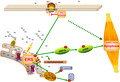

**Abbreviations**
4‐PBA: 4‐phenylbutyrate
CHOP: C/EBP‐homologous protein
CSA: cross‐sectional area
DM: differentiation medium
ER: endoplasmic reticulum
ERS: endoplasmic reticulum stress
GRP78: glucose‐regulated protein 78
MMP: mitochondria transmembrane potential
NNC‐55: NNC 55‐0396 hydrate
SR: sarcoplasmic reticulum
TA: tibialis anterior
TCC: T‐type calcium channel
TH: T‐type α1H channel
THKO: T‐type α1H channel knockout
ThT: thioflavin T
TUDAC: tauroursodeoxycholic acid

Skeletal muscle represents approximately 50–75% of the total body's proteins and is a highly dynamic and plastic organ that is responsible for generating the force [1]. Muscle wasting occurs systemically in many diseases conditions, including sarcopenia, cancer‐associated cachexia, sepsis, burns, and trauma [2]. Accumulating evidence indicates that the loss of skeletal muscle mass is associated with excessive apoptosis caused by a wide array of environmental and genetic factors [3, 4, 5]. However, its pathogenesis remains largely unclear.

Skeletal muscle contains a complicated network of the endoplasmic reticulum (ER) called the sarcoplasmic reticulum (SR), which is arranged in a dynamic tubular network. The ER is primarily recognized as the site of synthesis and folding of secreted proteins, serving as the site of Ca^2+^ uptake, storage, and release, which plays an important role in the regulation of proteostasis and calcium homeostasis in sophisticated scenarios [6, 7]. Altered ER homeostasis leads to the accumulation of unfolded or misfolded proteins in the ER lumen that may activate a homeostatic signaling network termed ER stress (ERS) [8]. ERS is buffered by activation of the unfolded protein response (UPR), which is initiated by three pivotal stress sensors [8, 9]. All three ERS sensors are maintained in an inactive state through their association with the ER chaperone glucose‐regulated protein 78 (GRP78) in resting cells and are activated by its dissociation [10]. Specific transcription factors are then activated, triggering the expression of UPR‐related genes [such as the proapoptotic transcription factor C/EBP‐homologous protein (CHOP)] [11, 12]. The role of CHOP in ERS‐induced apoptosis has been illustrated in *Chop* ^−/−^ mice [13]. Moreover, the activation of ERS signal cascades has been observed in skeletal muscle in multiple muscle‐wasting disease conditions with GRP78 upregulated [14, 15].

Emerging clinical, histopathological, and electrophysiological evidences suggest that alteration of T‐type Ca^2+^ channel (TCC) activity is likely to affect muscle afferent signaling and may be associated with amyotrophy and apoptosis sensitivity [16, 17]. There are three subtypes of TCC, and the T‐type α1H channel (TH) is the most significantly expressed [18]. A recent clinical study identified a loss‐of‐TH channel function because of *CACNA1H* variants is associated with infantile‐onset amyotrophy, perhaps through dysfunctional Ca^2+^ signaling [16]. Otherwise, the amplitude of T‐type Ca^2+^ currents has been found decreased significantly in aged skeletal muscle and aged satellite cells (SCs), resulting in inefficiency and the inability to fuse into myotubes during myogenic differentiation due to defective T‐type Ca^2+^ currents [19]. These results indicate that the function loss of TCC may play an important role in the pathogenesis of muscle wasting. On the basis of these studies, one new finding of cardiovascular diseases confirmed that both siRNA‐mediated gene knockdown and TCC blockers can induce ERS with relevance to cell viability [17]. Apart from the well‐established role of TCC blockade in cancer, cardiovascular diseases, and neurological disorders [20, 21, 22, 23], the consequences of TCC defects in skeletal muscle are still unknown.

We previously identified that the expression of TH (*CACNA1H*) is higher than that of the other two types in mammalian skeletal muscle and myoblasts. TCC inhibition by NNC 55‐0396 hydrate (NNC‐55) promoted ERS activation and autophagosome formation but blocked distal autophagy and autophagy flux [24]. Given that ERS is upstream of autophagy blockade induced by NNC‐55 and mitochondrial dysfunction could regulate autophagic process through ROS production and mitochondria‐specific proteins [25], we explore the primary effects of TCC function loss on ERS activation and mitochondrial‐related apoptosis. In this study, we found TCC inhibition induced ER‐Ca^2+^ disorder and ERS activation, which contributed to mitochondrial‐related apoptosis.

## Material and methods

### Cell culture and study design

C2C12 myoblasts were purchased from Cobioer Biosciences Co., Ltd. (Nanjing, China) and maintained in growth medium [Dulbecco's Modified Eagle Medium (DMEM) with 10% fetal bovine serum and 1% penicillin–streptomycin) in 5% CO_2_ at 37 °C for proliferation. For myogenesis, C2C12 myoblasts cultured in 6‐well plates were transferred into differentiation medium (DM; containing DMEM and 2% horse serum, 1% penicillin–streptomycin) for 4 days after reaching 80–90% confluence. The C2C12 myotubes were then treated with different drug interventions, respectively, for 24 h in DM for related analysis.

NNC‐55 (Sigma‐Aldrich Co, St. Louis, MO, USA), ERS inhibitors including sodium 4‐phenylbutyrate (4PBA, No.1716‐12‐7; MedChemExpress, Monmouth Junction, NJ, USA) and tauroursodeoxycholic acid (TUDAC, CAS No.35807‐85‐3, MedChemExpress), and intracellular Ca^2+^ chelator of BAPTA‐AM (CAS No.126150‐97‐8; MedChemExpress) were all dissolved in DMSO (Sigma‐Aldrich) and stored at −20 C or −80 °C.

For transfection, C2C12 myoblasts were transfected with negative‐control siRNA (NC) and three small interfering RNAs against TH including siRNA‐1, 2 and 3 (si‐1/si‐m‐Cacna1h‐001 5′‐GGGTAAACATCATGTACGA‐3′, si‐2/si‐m‐Cacna1h‐002 5′‐GGAATGTGGTTCTTTACAA‐3′ and si‐3/si‐m‐Cacna1h‐003 5′‐GTCGCATTGTAGACAGCA‐3′, Ribobio, Guangzhou, China) using riboFECT™CP Transfection Kit at a final concentration of 50 nm according to the manufacturer's protocol. After 48‐h transduction, the efficiency of TH knockdown was verified by quantitative real‐time PCR (qRT‐PCR), and the cells were cultured in DM for another 4 days to fuse into myotubes for subsequent analysis.

### Animals' management and sample

All experimental protocols of the animal study described here were approved by the Institutional Animal Care and Ethics Committee of the Renmin Hospital of Wuhan University (2016‐1105). All animal experiments comply with the ARRIVE guidelines and were carried out in accordance with the National Institutes of Health guide for the care and use of Laboratory animals. The TH knockout (THKO) mice were obtained from Jackson Laboratory (stock number 013770), and 4‐month‐old female mice were used for this experiment (*n* = 7). Female C57BL/6 mice, 4 months old, were obtained from the Experimental Animal Center of the Renmin Hospital of Wuhan University and used for a control group (*n* = 7). All mice were housed in a pathogen‐free barrier facility and were placed on a standard chow, *ad libitum*, in a temperature‐controlled room (21–22 °C) on a 12‐h light cycle for a week. All mice were sacrificed by cervical dislocation, and the left tibialis anterior (TA) muscles were harvested and fixed in 4% paraformaldehyde overnight, embedded in paraffin, and sectioned into muscle tissue sections, while right TA muscles preserved in −80 °C.

### Nuclear staining with Hoechst 333258

C2C12 myotubes that seeded onto glass coverslips in six‐well culture plate were fixed with 4% paraformaldehyde in PBS (Genom Biotech Ltd., Hangzhou, China) for 30 min and stained with Hoechst 333258 (Beyotime, China) for 20 min at room temperature. Then, cells were washed with PBS three times and examined under a fluorescence microscope (Olympus, Tokyo, Japan).

### Assays for mitochondria transmembrane potential by JC‐1 staining

The decrease of mitochondrial membrane potential is an iconic event in the early stages of apoptosis. The mitochondria with a lower membrane potential (∆Ψm) can be easily detected by the transformation of JC‐1 from red fluorescence(J‐aggregates) to green fluorescence (monomer). The mitochondrial transmembrane potential was assessed with a Mitochondrial Membrane Potential Assay Kit (Beyotime), termed JC‐1staining, according to the manufacturer's instructions. Briefly, add the CCCP (10 mm) recommended in the kit to the cell culture medium for 20 min as a positive control group. The indicated cells were loaded with 1× JC‐1 staining solution at 37 °C in the dark for 20 min and then washed, and a total of 20 000‐gated events analyzed by a flow cytometer (BD Biosciences, Franklin Lakes, NJ, USA). The JC‐1 fluorescence of red: green ratio was determined. The experiment was performed in triplicate.

### Mitochondrial superoxide production

Mitochondrial reactive oxygen species (mito‐ROS) production was measured using the MitoSOX™ Red mitochondrial superoxide indicator (40778ES50; Yeasen Biotech Co., Ltd., Shanghai, China) according to the manufacturer's instructions. DAPI was used as an indicator of nuclei. Cells were incubated with 5 μm MitoSOX™ Red solution for 15 min, washed three times, and mounted with DAPI, and images were then captured with a fluorescence microscope. Fluorescent intensity was analyzed byimagej analysis software (National Institutes of Health, Bethesda, MD, USA). In each experiment, more than 100 cells were investigated. The experiments were repeated three times.

### Intracellular Ca^2+^ detection in single C2C12 cell

The intracellular Ca^2+^ levels were measured using 10 µm Fluo‐3 AM (Sigma‐Aldrich). The treated and control cells were washed with Ca^2+^‐free Hank's balanced salt solution (HBSS, without phenolsulfonphthalein; Procell Life Science &Technology Co., Ltd., Wuhan, China) three times. After incubation with Fluo‐3 MA for 30 min at 37 °C with 5% CO_2_, cells were washed with HBSS three times. The fluorescence signals were detected in DM with a confocal laser scanning system (FV1200; Olympus Corp) by an argon laser at 488 nm and emitted a fluorescence beam at 530 nm, scanning the XY plane using the Times Course program. Images were acquired by continuous scanning to observe the fluorescence intensity changes of Ca^2+^ in each group, which was automatically plotted by LSCM image analysis system. Low concentration of KCl evoked increases of intracellular Ca^2+^ concentration by inducing Ca^2+^ influx. To estimate basal Ca^2+^ level, the average Ca^2+^ value for 1 min before stimulation was used. The increment of Ca^2+^ was calculated by subtraction of the basal level from each stimulus‐induced peak Ca^2+^ level (30 mm KCl). To quantify the effect of NNC‐55, basal Ca^2+^ level at rest and relative amplitude of Ca^2+^ induced by 30 mm KCl were calculated.

### Dye binding assays for ThT assay

Thioflavin T (ThT) is known to be able to detect ERS by exhibiting fluorescence when it binds to protein aggregates according to Beriault and Werstuck [26]. Briefly, cells were fixed in 4% paraformaldehyde, permeabilized by 0.1% Triton X/PBS, washed 3 times, and incubated with ThT (MedChemExpress) at the final concentration of 5 μm for 20 min at 37 °C. After washing three times with PBS, we mounted to slides using two drops of Fluoromount and stored at 4 °C in the dark. Confocal images were captured using an automatic microscope (BX63, Olympus Corp).

### Transmission electron microscopy analysis

Tibialis anterior muscles or cells were fixed in 2% glutaraldehyde (Servicebio, Inc., Wuhan, China) for 2 h. After a thorough rinsing in PBS, samples were post‐fixed in 1% osmium tetroxide and subsequently dehydrated with a graded series of ethanol. Samples were then embedded in Epon Resin 618. Ultrathin sections were obtained with a microtome, stained with uranyl acetate and lead citrate. Alterations in ER/SR and mitochondrial morphology were imaged using a Hitachi transmission electron microscope (HT7700; Hitachi, Ltd., Tokyo, Japan).

### Quantitative real‐time PCR

Total RNA was extracted from subjected cells using TRIzol reagent (Takara Bio Inc., Otsu, Japan) and then reverse‐transcribed into cDNA using the Hifair^TM^ II 1st Strand cDNA Synthesis Super Mix (gDNA digester plus; Yeasen Biotech Co., Ltd.). The primers for SERCA1, RyR1, IP3R1, GRP78, and CHOP were obtained from Yeasen Biotech Co., Ltd.. Primer sequences are listed in Table 1. The Hieff ^TM^ qPCR SYBR^®^ Green Master Mix (NO Rox Plus; Yeasen Biotech Co., Ltd.) reagent was used for qRT‐PCR, and the CFX96 Trademark Real‐time PCR detection system (Bio‐Rad, California, USA) was used for analysis. PCR thermal cycling conditions were 95 °C for 5 min, which could activate DNA polymerase, followed by 40 cycles (95 °C for 10 s, 56 °C for 20 s, and 72 °C for 20 s). Each experiment was performed in triplicates and repeated at least three times. The data of relative mRNA levels were calculated by the 2^−△△Cq^ method, and results were normalized to GAPDH expression and relative to expression in control.

**Table 1:** Primers for qRT‐PCR.

Gene	Forward primer(5′–3′)	Reverse primer(5′–3′)
SERCA1	5 ′ ‐CTCACACAAGTCCAAGATTGTG‐3 ′	5 ′ ‐GAGAAGTTATCATCGGCCAGTA‐3 ′
RyR1	5 ′ ‐CTGGGCTATGGCTACAACATC‐3 ′	5 ′ ‐GACTGCTTCAAACTCGAAGTAC‐3 ′
IP3R1	5 ′ ‐GTTTGAGAATTTCCTCGTGGAC‐3 ′	5 ′ ‐CATCACGATTTCAGTGACGTAC‐3 ′
GRP78	5 ′ ‐ATGATGAAGTTCACTGTGGTGG‐3 ′	5 ′ ‐CTGATCGTTGGCTATGATCTCC‐3 ′
CHOP	5 ′ ‐CTCCAGATTCCAGTCAGAGTTC‐3 ′	5 ′ ‐ACTCTGTTTCCGTTTCCTAGTT‐3 ′

### Immunofluorescence analysis

Immunofluorescent staining was performed for laminin (to demarcate the muscle fiber boundaries) and DAPI (to label the nuclei) co‐stained muscle images, and thus, muscle fiber size distribution can be plotted and analyzed by cross‐sectional area (CSA). Paraffin‑embedded TA muscle tissue sections were dewaxed by heating at 60˚C and washing in xylene (Sinopharm Chemical Reagent Co., Ltd., Shanghai, China) and rehydrated with a graded series of ethanol. Then, antigen retrieval was performed with microwave or citrate antigen retrieval solution followed by two washes with PBS and 1‐h incubation with blocking solution (5% BSA) prior to overnight incubation at 4 °C with rabbit polyclonal anti‐laminin primary antibody at 1 : 100 (Abcam, Cambridge, UK). Then, the tissue sections were incubated with the secondary antibody (Maxim Biotechnologies, Shanghai, China) labeled with FITC for 1 h at room temperature after washing extensively three times with PBS. Finally, the sections were mounted with DAPI and imaged with fluorescence microscope. Images were analyzed by ImageJ software (NIH, Bethesda, MD, USA).

### TUNEL analysis

Paraffin‑embedded sections of 5‐μm thickness were dewaxed by heating at 60˚C and washing in xylene (Sinopharm Chemical Reagent Co., Ltd.) and rehydrated with a graded series of ethanol. Then, in order to investigate apoptosis, terminal deoxynucleotidyl transferase‐mediated dUTP nick end labeling (TUNEL) analysis was performed on the muscle tissue sections using a commercially available TUNEL assay kit, in situ Cell Death Detection Kit, Fluorescein (Roche Diagnostics GmbH, Mannheim, Germany), following the manufacturer's protocol. The sections were incubated with a protease K working solution for 20 min at room temperature and then incubated with permeabilization solution for 10 min. After washing extensively three times with PBS, they were incubated with the TUNEL reaction mixture for 60 min at 37 °C in the dark. Finally, the sections were washed three times in PBS and imaged with fluorescence microscopy (IX51). The number of apoptotic cells and the total number of cells were calculated respectively.

### Western blot analysis

Tissue and cell extracts were homogenized in RIPA Lysis Buffer containing phenylmethylsulfonyl fluoride, centrifugated, and collected the supernatant to obtain whole cell lysates, and protein concentration was determined with the same way. Equal amounts of protein extracts were denatured by boiling at 100 °C for 8 min in SDS/PAGE Sample Loading Buffer. Next, the proteins were separated by 12% SDS/PAGE and then transferred to poly(vinylidene difluoride) membranes, blocked with 5% nonfat milk in Tris‐buffered saline with 0.05% Tween‐20 buffer (TBST) for 1 h at room temperature and incubated overnight at 4 °C with the following primary antibodies: rabbit antibodies against Bax (1 : 1000; Cell Signaling Technology Inc., Danvers, MA, USA), cleaved‐caspase3 (1 : 1000; Absin Bioscience Inc., Shanghai, China), Cytochrome c (Cyt‐c, 1 : 1000; Proteintech Group, Inc., Chicago, IL, USA), CHOP (1 : 1000 Proteintech Group, Inc.), Bcl2 (1 : 1000 Proteintech Group, Inc.), cleaved‐caspase 9 (1 : 1000; Cell Signaling Technology Inc.), β‐actin (1 : 3000; Proteintech Group, Inc.), and GRP78/BIP (1 : 3000; Proteintech Group, Inc.). The blots were washed with TBS‐T buffer three times and then incubated with horseradish peroxidase‐labeled goat anti‐rabbit IgG (H + L) secondary antibodies (1 : 3000; Servicebio, Inc.) for 1 h at room temperature. After washing with TBST, the blots were visualized using BeyoECL Star (Beyotime) and quantified using the Molecular Imager ChemiDoc Touch Imaging System with Image Lab 5.2 quantitative assay system (Bio‐Rad Laboratories, Inc., Hercules, CA, USA). The β‐actin protein was used as a control.

### Statistical analysis

All experimental data points are independent, and data are presented as the mean ± SD. Statistical software spss 17.0 (SPSS Inc., Chicago, IL, USA) was used for statistical analysis. For normally distributed data, one‑way analysis of variance (ANOVA) was used for multiple comparisons and Dunnett's test for comparing each group with the control group. Student's *t*‐tests (unpaired two‐tailed) were applied for comparing two groups. *P* < 0.05 was considered to indicate a statistically significant difference.

## Results

### TCC inhibition promotes apoptosis in C2C12 myotubes in a dose‐dependent manner

**Fig. 1:**
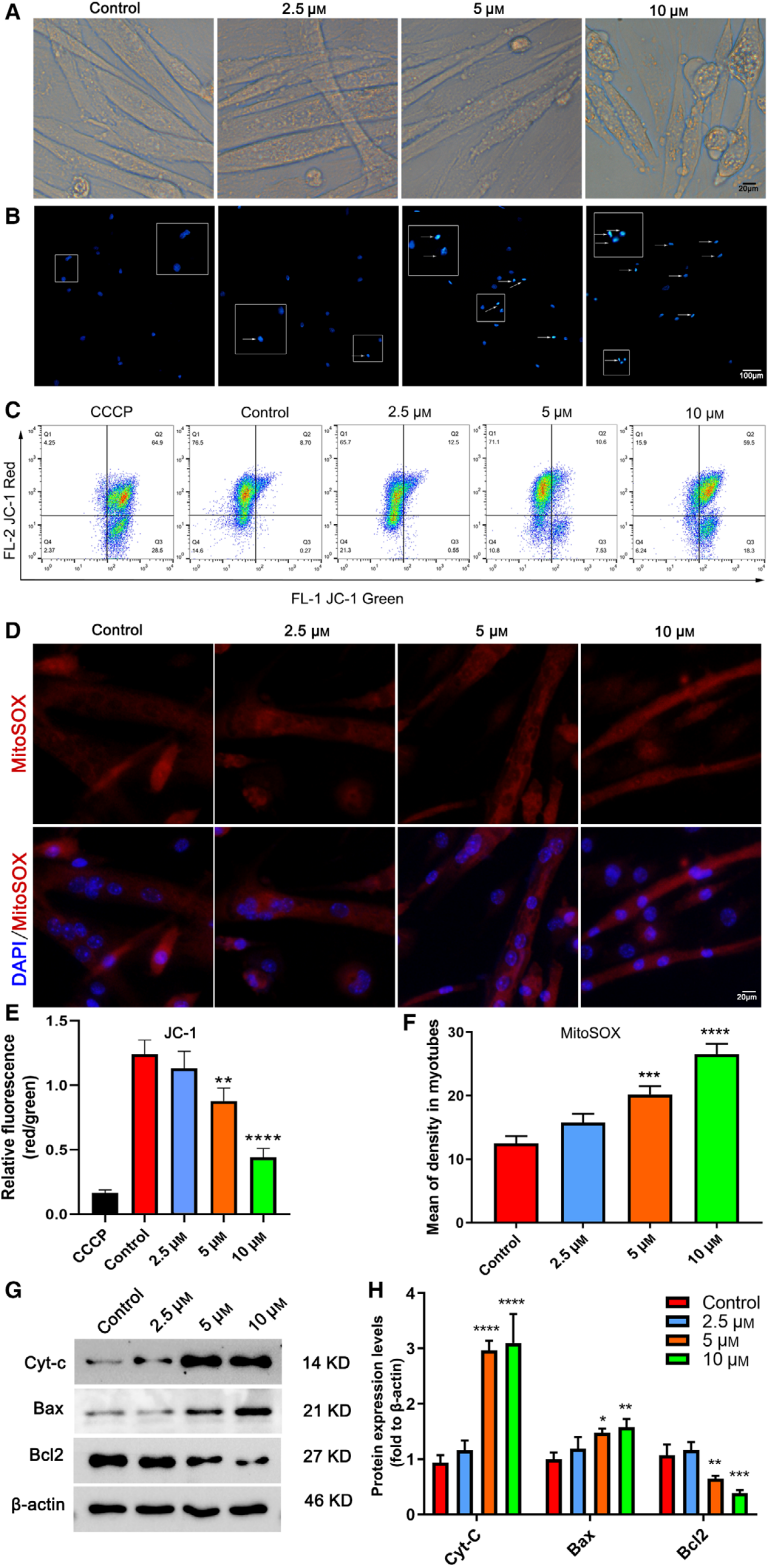
Apoptosis levels in myotubes with increasing concentration of NNC‐55 treatment for 24 h. C2C12 myoblasts were reseeded into six‐well plates and differentiated into myotubes for 4 days, followed by NNC‐55 treatment at various concentrations (0, 2.5, 5, and 10 µm; 0 µm was designated as the control group) for 24 h. Representative photographs of morphological changes of cell (A) and nuclei (typical of apoptosis, stained blue) stained with Hoechst 33258 (B). The apoptotic cells with strong fluorescence, fragmented, or condensed nuclei were observed under fluorescent microscopy, and selected fields illustrating occurrence of apoptosis were shown by white arrows. (C) JC‐1 flow cytometry analysis was performed, and the represented diagrams of flow cytometry were presented. (D) Representative imaging of mito‐ROS confocal images of each group and mean MitoSOX fluorescence intensities were presented. Quantitation of the JC‐1 red:JC‐1 green ratio in C2C12 myotubes treated by 5 and 10 μm NNC exhibited a decreased Δψm (E) and mean fluorescence density of MitoSOX in C2C12 myotubes (F). (G, H) Apoptosis‐related protein expression of C1C12 myotubes. Scale bars, 20 µm (A, D) and 100 µm (B); These data are presented as the (mean ± SD) for three independent experiments. *P < 0.05, **P < 0.01, ***P < 0.001, ****P < 0.0001 vs Control, respectively. One‐way ANOVA.

The morphology of NNC‐55‐treated C2C12 cells showed increased surface blebs, a cytological feature of C2C12 myotube apoptosis [27], and decreased myotube formation, which positively correlated with the concentration of NNC‐55 (Fig. 1A). This was further confirmed by analyzing the nuclear morphology that the apoptotic cells with strong fluorescence, fragmented, or condensed nuclei were observed under fluorescent microscopy, which showed that apoptosis increased in the same dose‐dependent manner (Fig. 1B). Similar results revealed that NNC‐55 treatment significantly reduced mitochondria transmembrane potential (MMP), increased mitochondria‐derived ROS levels, and expression of apoptotic protein (Cyt‐c, Bax upregulated, and Bcl2 downregulated) in myotubes (Fig. 1C–H), which are suggestive of apoptosis induction. These results suggested that pharmacological inhibition of TCC promoted apoptosis of C2C12 myotubes in a dose‐dependent manner.

### TCC inhibition increases ER‐Ca^2+^ release and interferes with ER Ca^2+^ homeostasis

**Fig. 2:**
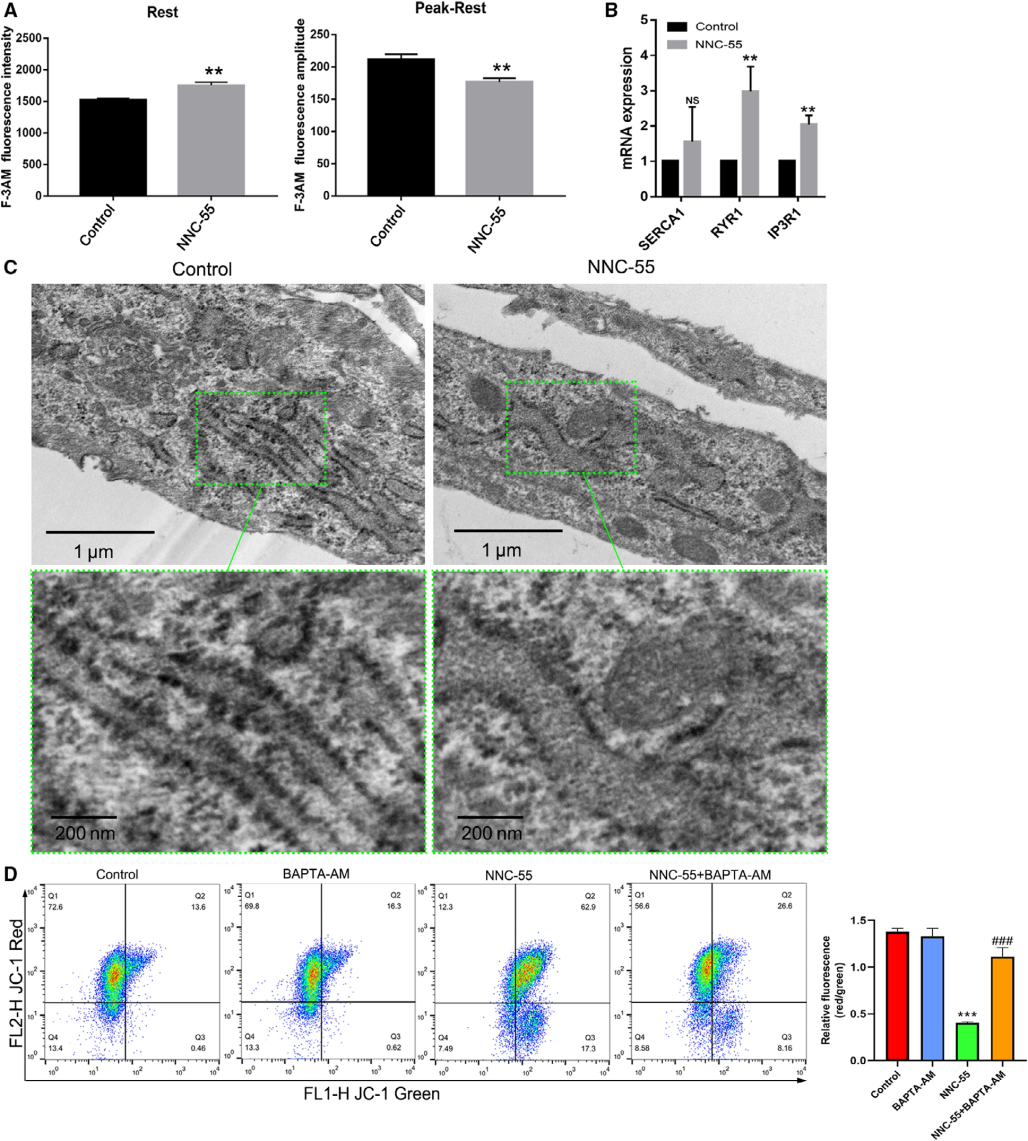
NNC‐55 treatment (10 μm, 24 h) induced ER‐Ca^2+^ release and ER‐Ca^2+^ dis‐homeostasis. (A) To estimate basal Ca^2+^ level, the average Ca^2+^ value for 1 min before stimulation was used. The increment of Ca^2+^ was calculated by subtraction of the basal level from each stimulus‐induced peak Ca^2+^ level (30 mm KCl). To quantify the effect of NNC‐55, basal Ca^2+^ level at rest and relative amplitude of Ca^2+^ induced by 30 mm KCl were calculated and the graphs presented the measurement of cytoplasmic Ca^2+^ concentration on a rest status and relative amplitude on a KCl‐induced peak‐rest status; (B) The gene expression related to important molecular components of the Ca^2+^ handling machinery of the ER (SERCA1, RYR1, IP3R1) by qRT‐PCR. (C) Morphological observations of subcellular structure in C2C12 myotubes treated by 10 µm NNC‐55 for 24h were obtained through transmission electron microscopy. Green frames indicate representative ultrastructural changes in top panels and were amplified in bottom panels. Scale bar, 1 µm (top panels) and 200µm (bottom panels). (D) MMP was assessed using JC‐1 staining by flow cytometry. JC‐1 fluorescence ratios (green/red) were calculated. JC‐1 staining showed that 10 µm NNC‐55 for 24 downregulated MMP with decreased JC‐1 red/green ratio in C2C12 myotubes compared to the control cells. These data are presented as the (mean ± SD) for three independent experiments. NS, no significance. **P < 0.01 vs Control; ***P < 0.001 vs Control; ^###^ *P* < 0.01 vs NNC‐55, respectively; One‐way ANOVA and Dunnett's test were applied.

Considering that apoptosis induction is associated with elevated intracellular Ca^2+^, a possible explanation for why C2C12 myotubes apoptosis increased after TCC inhibitor treatment may be pharmacological inhibitor‐mediated reduction in ER‐Ca^2+^ storage levels [28]. We examined the changes in intracellular Ca^2+^ levels after NNC‐55 treatment for 24 h using the fluorescent dye Fluo‐3 AM. It is known that low concentration of KCl evoked increases of intracellular Ca^2+^ concentration by inducing Ca^2+^ influx. We found intracellular Ca^2+^ started to increase after stimulation with 15 mm KCl and then peaked at 30 mm in C2C12 myotubes (data not shown), consistent with previous research [29]. As shown in Fig. 2A, the level of intracellular basal Ca^2+^ at rest was elevated, but the Ca^2+^ influx into cell at peak‐rest induced by 30 mm KCl in NNC‐55‐treated myotubes was less than that in control cells. These data indicated that the increased intracellular Ca^2+^ level is not due to the influx of extracellular Ca^2+^. Consequently, the mRNAs levels related to important molecular components of the Ca^2+^ handling machinery in the ER were upregulated. The results revealed that NNC‐55 treatment for 24 h increased ER‐Ca^2+^ release and induced dysregulation of ER‐Ca^2+^ homeostasis, as indicated by upregulated expression of SERCA1, RYR1, and IP3R1 (Fig. 2B). We also observed morphological changes in ER under transmission electron microscopy. Compared to the ER of the control, which had no swelling or expansion, the ER of the NNC‐55 group showed more edema, expansion, and vacuolation of the cavity and degranulation as noted by amplified green area (Fig. 2C). It should be noted that BAPTA‐AM, an intracellular Ca^2+^ chelator, markedly reversed NNC‐55‐induced MMP reduction, as evidenced by Fig. 2D. These results indicated that TCC inhibition increased ER‐Ca^2+^ release, resulting in interfered ER‐Ca^2+^ homeostasis and led to elevated intracellular Ca^2+^ levels in C2C12 myotubes and destruction of cellular organelle structures, which contributed to apoptosis.

### TCC inhibition promotes the activation of ERS‐related apoptosis in C2C12 myotubes

**Fig. 3:**
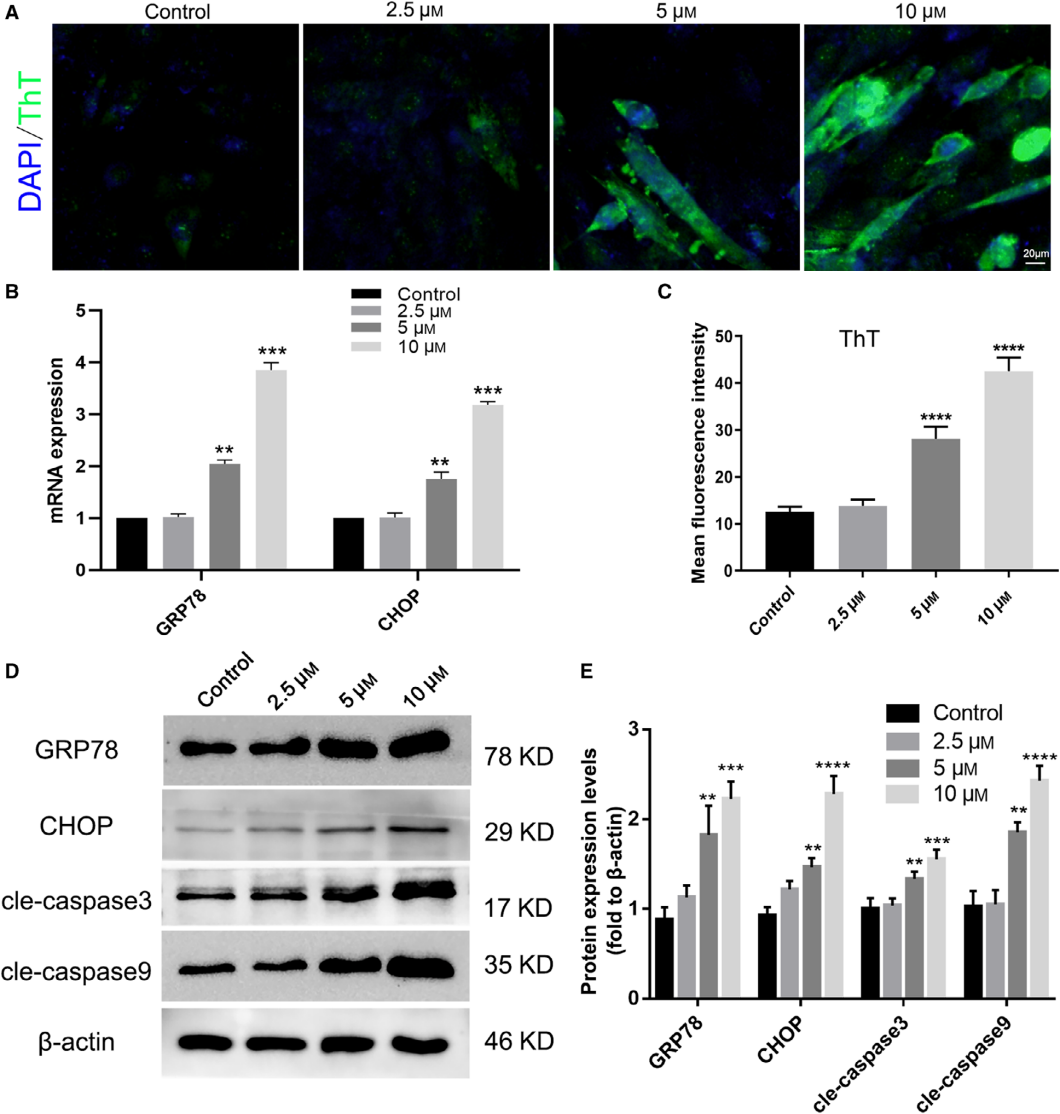
TCC inhibition induced ERS and increased apoptosis in C2C12 myotubes. C2C12 myotubes were treated with different concentrations of NNC‐55 after 4 days of differentiation according to the description in Fig. 1. (A) The intracellular ERS analysis by ThT assay under a fluorescence microscope and mean ThT fluorescence intensities were presented (C); ThT stained green and DAPI stained blue; scale bar, 20µm; (B) mRNA expression of ERS‐involved genes including GRP78 and CHOP by qRT‐PCR analysis in C2C12 myotubes. (D) Protein levels of GRP78, CHOP, cleaved‐caspase 3, and cleaved‐caspase 9 were analyzed by western blot and quantitation of proteins (E). These data are presented as the (mean ± SD) for three independent experiments. **P < 0.01, ***P < 0.001, ****P < 0.0001 vs Control, respectively. Data were analyzed using one‐way ANOVA and Dunnett's test.

Given that ERS‐mediated ER‐Ca^2+^ overload further exacerbates mitochondrial dysfunction and cell apoptosis [30], we detected ERS using ThT, which exhibits fluorescence when it binds to protein aggregates as Daniel described [26]. The results showed that the fluorescence intensity, which represents ERS levels, was significantly enhanced with the increase in NNC‐55 concentration in C2C12 myotubes (Fig. 3A,C). The mRNA expression of GRP78 and CHOP also upregulated, which positively correlated with the concentration of NNC‐55 (Fig. 3B). TCC inhibition significantly upregulated the expression of ERS activation‐related proteins (GRP78 and CHOP) and apoptosis‐related proteins (cleaved caspase 3 and cleaved caspase 9) in a concentration‐dependent manner (Fig. 3D,E). Together, these results indicated that TCC inhibition promoted the activation of ERS and apoptosis in C2C12 myotubes.

### ERS inhibitors attenuate the promotion of apoptosis and intracellular Ca^2+^ levels induced by NNC‐55

**Fig. 4:**
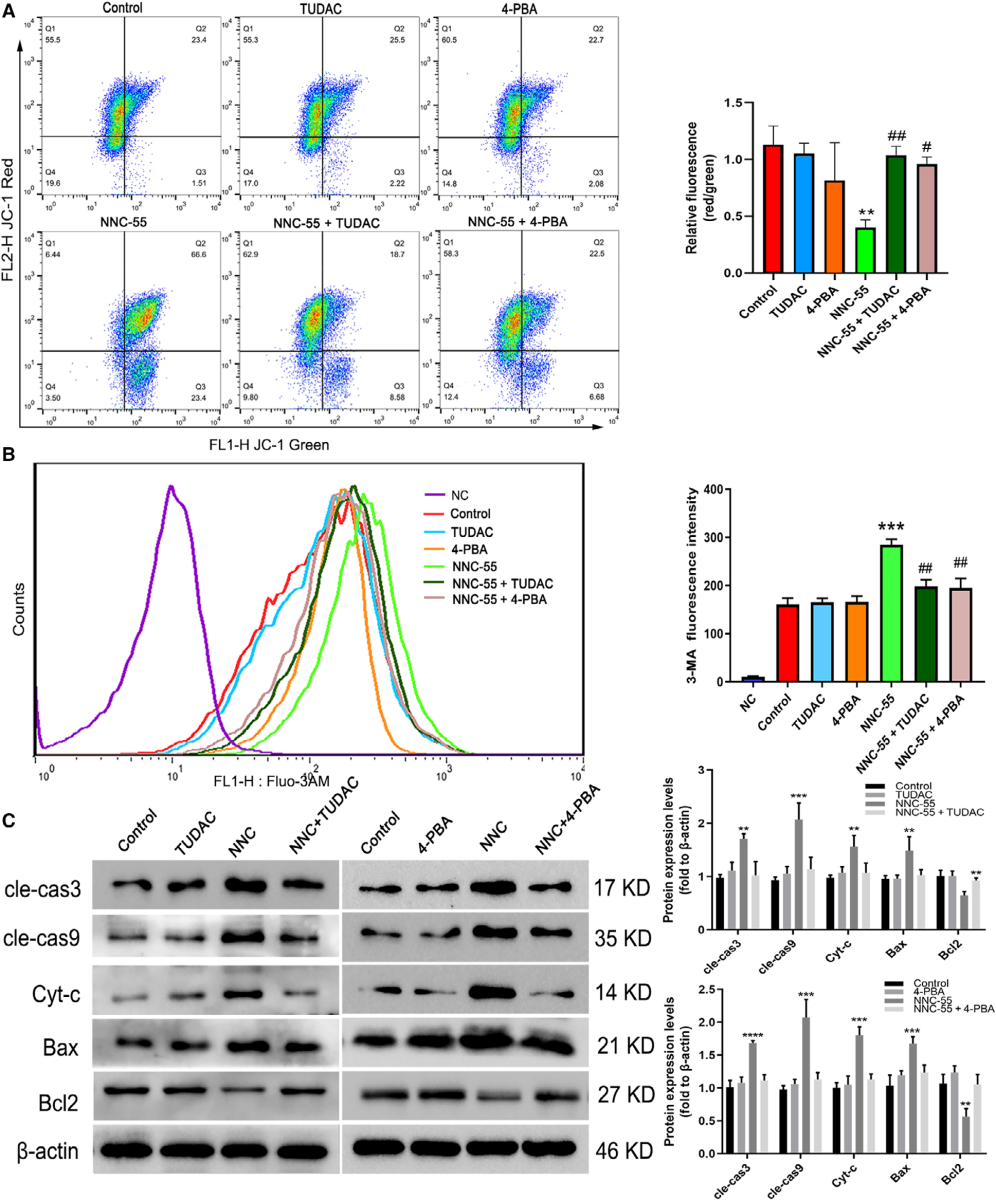
ERS inhibitors alleviated the promotion of apoptosis and cytoplasmic Ca^2+^ levels of C2C12 myotubes. After differentiated for 4 days, C2C12 myotubes were treated with different single‐time drug interventions for 24 h. (A) Measurements of JC‐1 red and green fluorescence were performed as described in Fig. 1. (B) Cytoplasmic Ca^2+^ concentration detection on rest status; (C) the protein expression and quantification of different intervention. These data are presented as the (mean ± SD) for three independent experiments. **P < 0.01 vs Control; ***P < 0.001 vs Control; ****P < 0.001 vs Control; ^#^ *P* < 0.05, ^##^ *P* < 0.01 vs NNC‐55. Data were analyzed using one‐way ANOVA and Dunnett's test.

To further investigate the contribution of ERS to mitochondria‐related apoptosis induced by NNC‐55 treatment, we reduced ERS with two ERS inhibitors, 4PBA [31] and TUDAC [30]. Interestingly, ERS inhibitors attenuated the promotion of MMP reduction induced by NNC‐55 (Fig. 4A). Moreover, the fluorescence intensity of intracellular Ca^2+^ staining by Fluo‐3 AM was significantly weakened with ERS inhibitor treatment, as shown in the two corresponding curves (Fig. 4B). The protein expression levels of cleaved caspase 3, cleaved caspase 9, Cyt‐c, and Bax were also downregulated after ERS inhibitor treatment; in particular, the antiapoptosis protein Bcl2 was significantly upregulated (Fig. 4C). The ERS inhibitors simultaneously attenuated the promotion of both mitochondria‐related apoptosis and intensity of Ca^2+^ in C2C12 myotubes induced by NNC‐55, suggesting a pathological mechanism in which apoptosis of C2C12 myotubes induced by TCC inhibition is ERS‐related.

### TH knockdown by siRNAs potentiates ERS and apoptosis in C2C12 myotubes

**Fig. 5:**
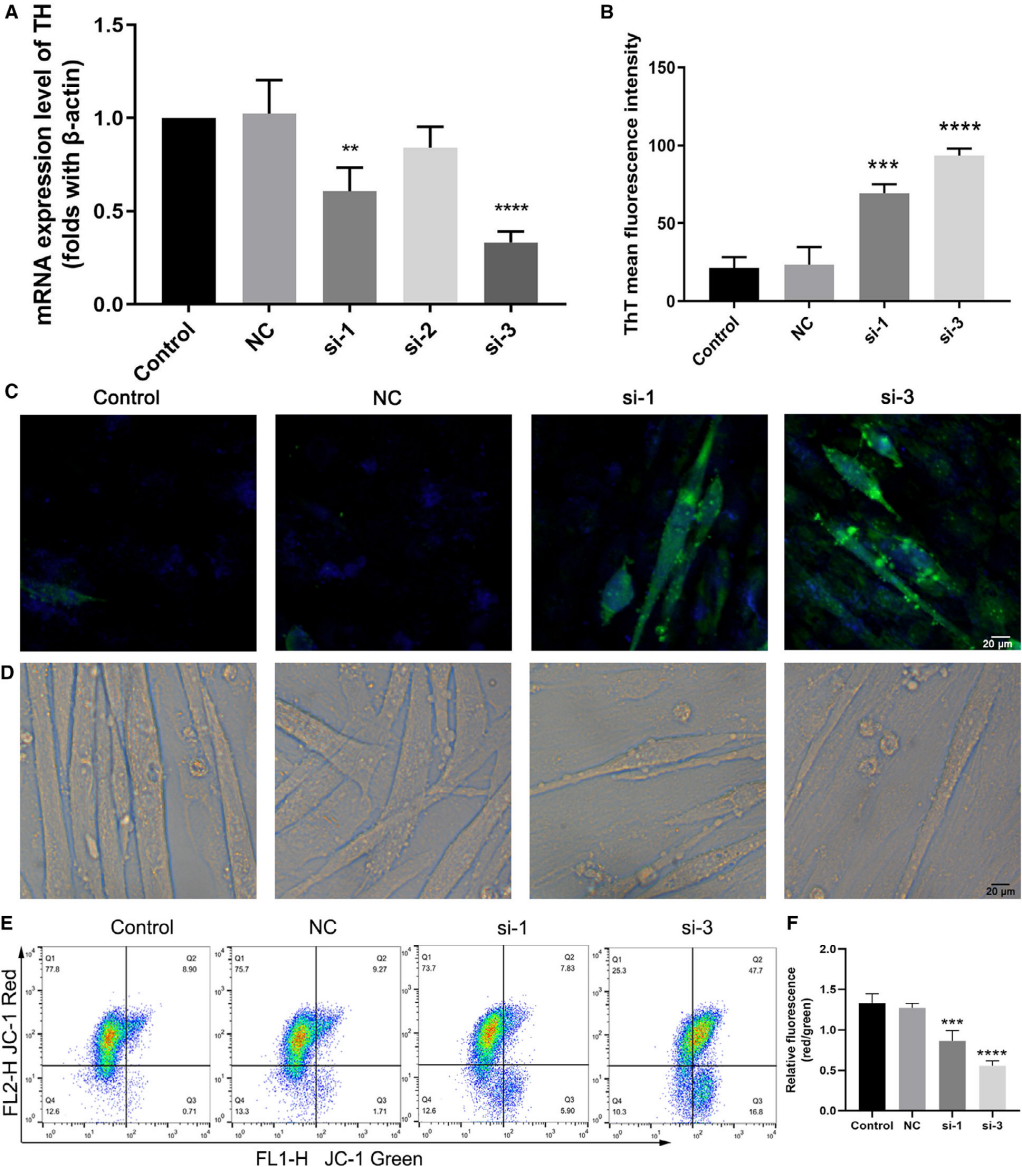
TH knockdown induced elevated ERS and apoptosis in C2C12 myotubes. (A) qRT‐PCR analysis of TH mRNA expression after TH silencing treatment for 48 h. (B, C) Representative ThT (green) fluorescence microscopic images and quantification, showing the ERS levels in myotubes. (D) Representative photographs of morphological changes of C2C12 myotubes; (E, F) MMP analysis using JC‐1 staining by flow cytometry and the relative fluorescence quantification of the JC‐1 red:JC‐1 green ratio as described in Fig. 1. These data are presented as the (mean ± SD) for three independent experiments. **P < 0.01, ***P < 0.001, ****P < 0.0001 vs Control. Data were analyzed using one‐way ANOVA and Dunnett's test.

To further verify the ERS‐related pro‐apoptotic effect of the TCC inhibitor on C2C12 myotubes, we generated TH knockdown C2C12 cells with three siRNAs. The expression of TH mRNA was mostly downregulated in the si‐1 and si‐3 groups (with a higher efficiency of TH silencing in the si‐3 group) compared to those of the control and NC groups (Fig. 5A). Consistent with the dose‐dependent effect of NNC‐55 treatment, we also found higher levels of ERS (Fig. 5B,C) and mitochondria‐related apoptosis in si‐3 group. The morphology of TH‐silenced C2C12 myotubes showed more surface blebs and decreased myotube fusion in the si‐1 and si‐3 groups (Fig. 5D). In addition, a significant decrease of MMP was found in the si‐1 and si‐3 group (Fig. 5E,F). These results further verified that TCC blockade caused by TH knockdown has a significant effect on ERS activation and promoting mitochondria‐related apoptosis in C2C12 myotubes.

### TH knockout mice display muscle wasting with reduced skeletal muscle fiber size and increased ERS‐related apoptosis levels

**Fig. 6:**
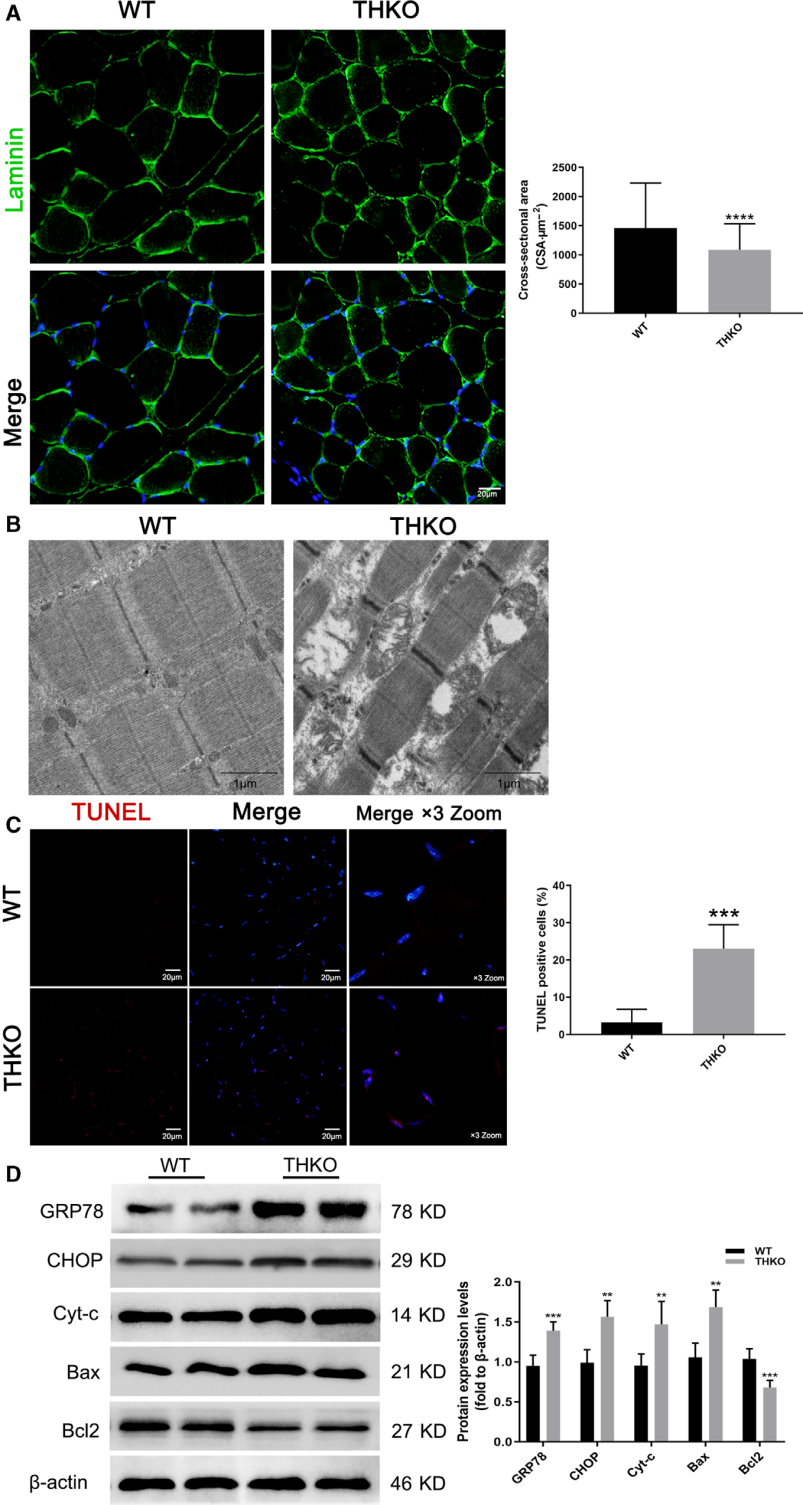
THKO mice displayed smaller muscle fiber size and ERS‐related apoptosis levels in TA. (A) CSA of TA muscle using laminin (green) and merged DAPI (blue) staining, and the quantification. Scale bar, 20 µm; (B) the ultrastructural morphology observation by a transmission electron microscope. Scale bars, 1 µm. (C) Cell apoptosis in TA muscle tissue by TUNEL assay. TUNEL‐positive cells were stained red, and cell nuclei were stained blue by DAPI. Scale bars, 20 µm; the merged images were amplified three times; (D) western blot results for GRP78, CHOP, Cyt‐c, Bax, and Bcl2 expression. These data are presented as the (mean ± SD) for three independent experiments. **P < 0.01, ***P < 0.001, ****P < 0.0001 vs Control by Student's *t*‐tests (unpaired two‐tailed).

As a proof‐of‐principle, we then sought to validate the physiological relevance of TCC blockade to skeletal muscle mass and its associated signaling molecule(s) related to apoptosis *in vivo*. To address this, we subjected TA muscles from wild‐type (WT) and THKO mice to immunoblotting and ultra‐microstructure analyses. Although we did not find an abnormal phenotype in myogenic development, THKO mice displayed muscle wasting. As quantified by CSA, THKO mice displayed a smaller muscle fiber size (Fig. 6A). In addition, THKO muscle sections showed major ultrastructural defects, including loosely packed and disrupted muscle bundles, impaired ER with degranulation in cisternae, as well as swollen mitochondria, whereas WT samples exhibited normal ultrastructure and a tidal structure in the SR zone (Fig. 6B). Furthermore, the percentage of red TUNEL‑positive cells was significantly increased in the THKO muscles, indicating significantly elevated apoptosis in THKO muscle (Fig. 6C). Accordingly, THKO muscles showed a uniform increase in the expression of both ERS‐related (GRP78 and CHOP) and apoptotic proteins (Cyt‐c and Bax), along with significant downregulation of an antiapoptotic protein (Bcl2; Fig. 6D). Together, we concluded that TCC blockade significantly contributes to the occurrence of ERS and apoptosis in skeletal muscle, which is proposed to be one of the mechanisms underlying muscle wasting.

## Discussion

Skeletal muscle is a highly dynamic tissue and undergoes continuous remodeling that is responsible for cellular stress. Loss of muscle mass can be the consequence of pathological changes, as observed in muscular dystrophies; the chronic muscle disuse that accompanies aging or muscle‐wasting diseases provokes a decline in mitochondrial content and function, which elicits excessive ROS formation and apoptotic signaling [32]. ER proteostasis surveillance mediated by ERS also plays a crucial physiological role in the maintenance of skeletal muscle and is emerging as a possible driver of cell apoptosis. Our previous study uncovered that TCC inhibition induced C2C12 myotube atrophy through ERS activation and autophagy flux blockade [24]. ERS is upstream of autophagy blockade induced by NNC‐55, and mitochondrial dysfunction could also regulate autophagic process through ROS production and mitochondria‐specific proteins. Therefore, we further explored the effects of TCC function loss on mitochondria‐related apoptosis which may be a trigger of autophagy regulation. Here, we confirmed a novel mechanism that TCC blockade resulted in the occurrence of ERS through CHOP‐mediated impingement on the regulation of mitochondria‐related apoptosis, which may be involved in the development of muscle wasting.

T‐type calcium channel, which is activated by low voltage, is expressed ubiquitously in multiple cell types and organs and mediates intracellular Ca^2+^ signal transduction [23]. As aged skeletal muscle and aged SCs showed decreased amplitude of T‐type Ca^2+^ currents[19], it can be speculated that TCCs may alter or blockade with aging. In addition, as an aging‐specific response of age‐related declines in muscle mass (sarcopenia) [14], the activation of ERS signal cascades has been observed in skeletal muscle in multiple muscle‐wasting conditions with GRP78 upregulated. ERS is stimulated by a wide range of cellular environments and events, including increased levels of protein synthesis, an excess or limitation of nutrients, inflammatory challenges, and dysregulated Ca^2+^ levels [9]. Accordingly, we hypothesized that TCC blockade acts as one of the triggers that alters ER homeostasis and initiates the activation of GRP78. We found that the TCC inhibition induced apoptosis and promotes ERS in C2C12 myotubes in a dose‐dependent manner and THKO muscle displayed muscle wasting with smaller myofiber size (Fig. 6A). These results suggested effects of apoptotic cell death and ERS induction by TCC blockade.

Considering that ERS‐mediated ER Ca^2+^ overload further exacerbates mitochondrial dysfunction and cell apoptosis [30], a possible explanation for why C2C12 apoptosis increased after TCC inhibition may be ERS‐mediated reduction in ER Ca^2+^ storage levels. The maintenance of internal Ca^2+^ homeostasis is an essential feature of the proper structure and physiological function of skeletal muscle and is critically required for maintaining homeostatic conditions and structural integrity in the ER/SR zone [18]. Thus, in addition to ER impairments characterized as obvious degranulation and dilation in cisternae in the ER zone (Fig. 3B), We found that TCC inhibition induced interfered ER‐Ca^2+^ homeostasis (Fig. 2B) and increased intracellular Ca^2+^ levels. As a proof, intracellular Ca^2+^ chelator markedly reversed NNC‐55‐induced apoptosis and ERS inhibitors attenuated the promotion of both apoptosis and intracellular Ca^2+^ levels (Fig. 2C). Since persistent ERS induces downregulation of total and ER‐Ca^2+^ stores, it has been reported that ERS increases mitochondrial Ca^2+^ uptake and ROS production, which contributes to decreased mitochondrial transmembrane potential (∆Ψm) [28, 31, 33, 34] and promotes cell survival or cell death. It is reasonable that the close distance associated with the flux of Ca^2+^ between the ER and mitochondria may couple with increased Ca^2+^ uptake by mitochondria at MCSs. These data suggest that apoptosis induction is associated with ERS‐related ER‐Ca^2+^ dis‐homeostasis.

It has been confirmed that CHOP is controlled by the PERK–ATF4 axis when ERS sensors are activated under certain conditions, which induces the expression of proapoptotic genes [12]. CHOP has also been shown to induce apoptosis through the core mitochondrial apoptosis pathway, which is regulated by members of the B cell lymphoma 2 family (i.e., Bax and Bcl2) and ultimately leads to caspase activation [8, 35]. Consistent with this, our data showed increased percentage of red TUNEL‑positive cells and a significant upregulation of GRP78, CHOP, Cyt‐c, and Bax and downregulation of Bcl2 in THKO muscle (Fig. 6D). These results can be explained by the mechanism of which TCC blockade resulted in the occurrence of ERS through CHOP‐mediated signal pathway. However, more further studies on detailed intermolecular pathways are required to clarify the role of TCC blockade in skeletal muscle.

In conclusion, our data highlight the mechanism by which TCC blockade induces the occurrence of ERS through CHOP‐mediated impingement on the regulation of mitochondria‐related apoptosis. ERS‐induced ER Ca^2+^ disorders and increased intracellular Ca^2+^ levels contribute to ER impairments and proapoptotic gene expression, which may be involved in the development of skeletal muscle wasting.

## Conflict of interest

The authors declare no conflict of interest.

## Author contributions

MC and LH conceived and supervised the study; MC and SL designed experiments; MH, JC, and ZZ performed experiments; SH provided new tools and reagents; SH, JM, and JT developed new software and performed simulation studies; MH analyzed data; MC wrote the manuscript; and LH made manuscript revisions. All authors read and approved the final manuscript.

## Data Availability

All data used to support the findings of this study are included within the article.
